# South Africa’s Health Promotion Levy on pricing and acquisition of beverages in small stores and supermarkets

**DOI:** 10.1017/S1368980022000507

**Published:** 2022-05

**Authors:** Alexandra Ross, Elizabeth C Swart, Tamryn Frank, Caitlin M Lowery, Shu Wen Ng

**Affiliations:** 1 Department of Nutrition, Gillings School of Global Public Health and Carolina Population Center, University of North Carolina, Chapel Hill, NC 27599-8120, USA; 2 Department of Dietetics and Nutrition, University of the Western Cape, Cape Town, Republic of South Africa; 3 DST/NRF Center of Excellence in Food Security, University of the Western Cape, Cape Town, Republic of South Africa; 4 School of Public Health, University of the Western Cape, Cape Town, Republic of South Africa

**Keywords:** Food environment, SSB tax, South Africa, Food policy, Sugar-sweetened beverage

## Abstract

**Objective:**

In response to concern over rising sugar-sweetened beverage (SSB) consumption, in April 2018, South Africa became the first Sub-Saharan African (SSA) country to implement an SSB tax. We assess changes in pricing and acquisition of beverages from local supermarkets and small stores among 18–39-year-old adults living in one township in the Western Cape, before and after tax implementation. This study is among the first evaluations of an SSB tax on the local food environment in a low-income township.

**Design:**

Store beverage pricing and participant surveys were cross-sectional, analysed 1 month before and 11 months after implementation of the tax (March 2018 and March 2019).

**Setting:**

Langa, Western Cape, South Africa

**Participants:**

Surveyed participants were residents of Langa between 18 and 39 years old (*n* 2693 in 2018 and *n* 2520 in 2019)

**Results:**

Prices of taxed SSB increased significantly among small shops and supermarkets between 2018 and 2019. There were non-significant decreases in prices of untaxed beverages in small shops, but prices of untaxed beverages increased in supermarkets. Across all store types, there was a 9 percentage point decrease in the probability of purchasing regular soda weekly pre/post-implementation. Reductions in purchasing were larger in small shops than supermarkets.

**Conclusions:**

We found some differential impacts of the levy on pricing and acquisition of beverages by retailer type in one low-income township. As other SSA countries consider similar fiscal policies to curb soda consumption, obesity and related diseases, this work can be used to understand the implications of these policies in the retail setting.

Excessive consumption of sugar-sweetened beverages (SSB) is strongly associated with weight gain, type 2 diabetes mellitus and other metabolic conditions^(1–5)^. Nevertheless, consumption of sugary drinks remains high globally, including in South Africa, where the rates of consumption have grown in both urban and rural areas^(5–9)^. Sub-Saharan Africa (SSA) faces increasing rates of diet-related non-communicable diseases with rapidly rising intakes of SSB and other ultra-processed foods^(10–13)^. Similarly, studies show that overweight and obesity are increasing in SSA, with faster increases in urban areas^(14,15)^. The burdens of obesity and related non-communicable diseases such as diabetes, hypertension and some cancers are growing in South Africa, where almost 40 % of women and 11 % of men are obese^(16–19)^.

In urban South Africa, food trade is the basis for more than one-third of township informal business and plays an important role in making affordable food locally accessible^(20–22)^. Small independently owned shops, known as ‘spazas’ referred to as ‘small shops’ in this paper, and traditional food vendors in townships still dominate the food system, and often have limited depth of stock and refrigeration to be able to store fresh foods^(9)^. Given the dominance of these small shops in the local food system, it is likely that a major portion of ultra-processed food and SSB are purchased in small shops. At the same time, supermarkets are increasing in South Africa, specifically in low-income settings, which has the potential to increase food access in a country that is characterised by a double burden of malnutrition^(23–25)^. It is often the case that residents that are employed and have their own transport are likely to shop at supermarkets near their place of employment or will drive to large supermarkets for their monthly grocery shopping, and spazas are used to procure single items in between trips to supermarkets. Supermarkets have more variety per category than small shops, but both retail venues sell the same categories of sugary, salty and oily ultra-processed foods and beverages^(26–28)^.

In response to the increasing concern over the rising consumption of ultra-processed foods, specifically SSB, in April 2018, South Africa became the first SSA country to enact an SSB tax. This excise tax, called the Health Promotion Levy (HPL), was a tiered sugar-based excise tax implemented at a rate of 0·021 ZAR for each gram of sugar over an initial threshold of 4 g/100 ml. Policies like the HPL have the potential to reduce overconsumption of cheap, ultra-processed foods which are high in nutrients shown to be linked with poor health outcomes (termed ‘unhealthy’ henceforth) with policy actions and (dis)incentives. Policies such as SSB taxes, mandatory warning labels and restrictions on marketing/advertising of unhealthy foods have the potential to balance out the retail food environment in this regard^(9,29)^.

It is essential to understand how SSB taxes impact low-income food environments, as low-income individuals may be at higher risk for diet-related non-communicable diseases and have less access to healthcare. In particular, it is important to understand how the tax differentially impacted pricing and purchasing in the venues that sell them most: supermarkets and small shops. One of the intentions of the SSB tax is to decrease purchasing and lower sugar intake. A limitation of prior studies that incorporate datasets that assess purchasing and pricing, such as Kantar World Panel, is that the source population is often on average higher income than the general population and thus misses a large share of lower income purchases from retailers like small shops^(30)^. In order to address this limitation, this study focuses on a low-income population and represents one of the first evaluations of an SSB tax on the local food environment in a low-income township.

As part of an evaluation of the HPL, surveys were conducted in Langa, Western Cape, South Africa between February 2018and March 2019, before and after implementation in April 2018. Previous studies have identified reductions in sugar consumption using dietary recalls in this context, as well as changes in purchasing from national panel data^(30–32)^. Outside of South Africa, most evaluations of the impact of SSB taxes on prices and purchasing have been conducted in higher income countries or in higher income communities^(33–36)^. There is potential for heterogeneity in response to SSB taxes for different subpopulations based on socio-economic status, age and baseline level of SSB consumption. Specifically, evidence from other countries has shown that lower income households are more responsive to taxes, whilst higher SSB purchasing households reduce their amount of SSB purchases more^(36–38)^, which if sustained can be clinically meaningful. Additionally, the long-term health benefits of reducing SSB consumption are larger for younger adults, and particularly for high SSB consumers.

To address the existing research gaps related to the impact of an SSB tax on low-income and often overlooked subpopulations, the aim of this study is to assess changes in pricing and acquisition of beverages from local food stores among 18–39-year-old adults living in one low-income township in the Western Cape, before and after the HPL implementation in April 2018. An understanding of food retail and consumer responses to the tax in low-income communities with a mixture of formal and informal food retail may inform low and middle-income countries, particularly in SSA that are battling the double burden of malnutrition and considering similar fiscal policies to South Africa’s sugary beverage tax.

## Methods

### Setting

This study was conducted in Langa, a township located in Cape Town, in the Western Cape. Our study population was selected due to the stability of the Langa community for repeated data collection across time. At last count (in 2011), Langa had 17 402 households and 52 401 inhabitants (50·4 % female), of whom 99·1 % were of Black African race^(39)^. From observation of the area during data collection from 2018 to 2019, 1 supermarket, 72 small shops, 43 street vendors, 14 family grocery or convenience stores, 6 sit-down food services and 37 cooked food outlets (including roadside outlets) were identified in Langa.

### Pricing data

Pricing data were collected to understand the cost of beverages in retail outlets most used by consumers in this urban area, and how prices changed post-implementation of the HPL. Fieldworkers traveled to stores and documented the prices of 2362 commonly stocked taxed and untaxed beverages that were stocked across all 7 time points: March 2018 (pre-tax), April 2018 (immediately after implementation), May 2018, October 2018 (6 months after implementation), March 2019 (1-year post-implementation), July 2019 and October 2019. Only the March 2018 and March 2019 timepoints are included in the data analysis. The same supermarkets and small shops participated in every point of data collection. All 19 small shops were located within Langa. The 5 supermarket locations from which prices were collected represent the 4 largest retail chains in South Africa. One supermarket is located in Langa, and 4 in Durbanville, Cape Town, which is approximately 30 min away from Langa by car. Price was collected with specificity to brand, flavour and package sizing. Prices were converted to South African Rand per liter (ZAR/L) for the analysis. Taxed beverages included regular sodas, concentrates, juice drinks (not 100 % juice), sports drinks, energy drinks, flavoured waters, prepackaged sweetened coffees and teas with added sugar > 4 g/100 ml, drinking yogurt and flavoured milk. Untaxed beverages included carbonated and still bottled waters, sugar-free diet sodas, coffee and tea with < 4 g of sugar/100 ml), 100 % juice, as well as fresh and shelf-stable milk.

### Acquisition data

One objective of this work is to understand the change in frequency of beverages purchased by lower income adult consumers in this urban area of South Africa before and after the implementation of the HPL. Survey participants were between 18 and 39 years old and living in Langa. Surveys included anthropometric measures, a household questionnaire assessing food acquisition, knowledge, attitudes, perceptions and behaviours and a 24-hour diet recall and beverage questionnaire. Data were collected in February–March 2018 (pre-HPL implementation; *n* 2693), and in February–March 2019 1 year following (*n* 2520) to measure differences in acquisition following implementation. Participants were asked how often they purchased dairy, other dairy products (including dry milks), regular soda, diet soda and coffee/tea (never, daily, 2–3×/week, weekly, fortnightly, monthly, special occasion). Participants were also asked where they usually purchased each beverage type: at wholesalers, supermarkets, small shops/informal convenience stores or other independent retailers. ‘Other independent retailers’ include house shops, market traders, container shops, bakkie traders, etc. To record anthropometry and BMI, fieldworkers used standardised scales and stadiometers to record the weight and height of each participant after the diet recall was completed, measuring each twice.

Systematic door-to-door sampling was conducted in February–March 2018 and February–March 2019. One randomly selected consenting adult between the ages of 18–39-year-olds per household was included in the study. The interviewer-administered household questionnaire was completed digitally using android phones and included a geolocation. The geolocations provided us with maps to ensure that all areas were covered in the sampling strategy. Digital data collection was performed offline and then uploaded and synced later. Participants received a supermarket voucher worth R30 (USD$ 2·19) after participating. During post-tax data collection, individuals were asked whether they participated in the previous round of survey. However, the data are not longitudinal because observations cannot be linked across time, so the data is treated as a repeated cross-sectional study. Also, while pricing and acquisition data were collected in the same geographical area, it was not possible to link them to each other. Most respondents who reported shopping at small shops likely shopped at small shops within Langa, but we are not able to know which shops specifically. Additionally, although there was only one supermarket in Langa, if a participant reported purchasing a product from a supermarket, it could be a supermarket in another area as people may shop where they work or occasionally purposefully travel to another location for the purpose of shopping. All participants gave written informed consent to enroll in the study.

### Statistical analysis

All analyses were conducted in Stata, version 16^(40)^. We analysed each type of data (pricing and acquisition) separately since the units of measurements were different.

### Pricing data

Pricing of SSB was compared by volume (multi-serve (> 500 ml) *v*. single serve), tax status (taxed *v*. untaxed) and store type (supermarkets *v*. small shops). Beverages were coded as taxed or untaxed based on the threshold defined in the regulation and the sugar content of the beverage (above or below the threshold of 4 g of sugar/100 ml). Milk and 100 % fruit juice are exempt. To account for seasonality and for alignment with the acquisition analyses, pre/post analyses compared prices from the same months: March 2018 and March 2019. Paired *t* tests were performed to compare differences in price between pre-tax and 1-year post-tax observations. Our key outcome was a change in price per litre (ZAR/L) for taxed beverages and untaxed beverages.

### Acquisition data

This analysis examined differences in the frequency of beverage purchases, including milk, diet soda and regular sodas in the post-tax period compared with pre-tax period. Models were adjusted for age (continuous, range 18–39), sex, weight status (categorical based on BMI derived from measured height and weight), number of children in the household, number of people in the household, beverage consumption and socio-economic status. Socio-economic status was ascertained using the South African Audience Research Foundation’s Living Standards Measure. It is based on 29 items, including household income and education and household assets^(41)^. Most of the study sample was at or below an Living Standards Measure category of 6, based on a range of 1–10 (Table 1). Our sample data collected in the pre-period was collected during a drought period in the Western Cape between January and June 2018 and strict water controls were enforced. We tested whether our results were confounded by water shortages by controlling for a variable on perceived change in beverage consumption due to the drought and water availability in and around the home. Other covariates included lived poverty index measures relating to the availability of food and water, and psychosocial measures relating to awareness of the HPL. We used logistic regression to estimate differences in the probability of weekly beverage acquisition in the post-tax period compared with pre-tax period. Models were adjusted for the covariates listed above. The Stata margins command was used to estimate and interpret adjusted predictions and marginal effects.

**Table 1:** Characteristics of residents (age 18–39 years) living in Langa, Western Cape, South Africa, 2018–2019

	2018 (*n* 2693)			2019 (*n* 2520)		
Age	Mean = 27·87, sd = 5·9			Mean = 27·74, sd = 6·2		
	*n*	%	Missing (*n*)	*n*	%	Missing (*n*)
Gender			134			35
Male	895	34·9		859	34·6	
Female	1664	65·0		1626	65·4	
LSM category*			542			76
2	86	4·0		0	0·0	
3	37	1·7		49	2·0	
4	322	14·9		477	19·5	
5	847	39·4		1240	50·7	
6	859	39·9		678	27·7	
BMI			672			388
< 18·5	107	5·3		105	4·9	
18·5–24·9	874	43·3		925	43·4	
25·0–29·9	490	24·3		502	23·6	
> 30·0	550	27·2		600	28·1	
Reported consumption of sugar-sweetened beverage in 24-h recall	1237	45·9	0	1016	40·3	0

* Based on a range of 1–10.

## Results

### Pricing

Across both store types and tax status, the price differences (ZAR/L) were higher for single-serving beverages than multi-serving (greater than 500 ml) beverages (Table 2). We found statistically significant increases in prices of taxed beverages in small shops (+1·83 ZAR/L for multi-serving beverages, *P* < 0·01; and +3·03 ZAR/L for single-serving beverages, *P* < 0·01) after implementation of the HPL in 2019. The increase in taxed beverage prices was similar across store types (with wider confidence intervals for the supermarkets likely due to smaller sample sizes), but the change in untaxed beverage price estimates differs. There was a non-significant decrease of 1·86 ZAR/L in prices for untaxed beverages in spazas (*P* = 0·15), but an increase of 4·86 ZAR/L in prices of untaxed beverages in supermarkets (*P* = 0·02).

**Table 2:** One-year change in beverage prices (ZAR/L) by store type and volume in Western Cape, South Africa March 2018–March 2019

	Pre-tax (March 2018)		Post-tax (March 2019)		Difference (post–pre)		
	Mean (ZAR/L)	95 % CI	Mean (ZAR/L)	95 % CI	Mean (ZAR/L)	95 % CI	pval
19 independent convenience stores ‘Spazas’							
Untaxed	14·68	13·56, 15·80	13·56	12·59, 14·53	−1·12	−2·62, 0·38	0·14
Multi-serve*	12·34	11·44, 13·24	11·93	11·07, 12·80	–0·41	−1·65, 0·84	0·52
Single serve	20·94	18·95, 22·93	19·08	17·66, 20·50	−1·86	−4·44, 0·73	0·15
Taxed	14·54	13·89, 15·20	16·41	15·46, 17·36	1·87	0·75, 2·98	0·001
Multi-serve	9·16	8·77, 9·55	10·99	10·39, 11·59	1·83	1·15, 2·51	0·0000
Single serve	21·48	20·53, 22·42	24·51	22·96, 26·06	3·03	1·32, 4·74	0·0005
Five large chain supermarkets							
Untaxed	22·93	21·65, 24·21	27·11	24·73, 29·49	4·18	1·64, 6·72	0·001
Multi-serve	16·34	15·29, 17·39	18·16	15·90, 20·42	1·82	–0·41, 4·04	0·10
Single serve	31·75	29·50, 33·99	36·61	33·05, 40·17	4·86	0·71, 9·00	0·02
Taxed	29·02	27·36, 30·68	31·66	29·89, 33·43	2·64	0·09, 5·20	0·04
Multi-serve	15·90	14·50, 17·29	17·79	15·60, 20·00	1·90	–0·57, 4·37	0·13
Single serve	35·13	32·99, 37·27	38·08	36·13, 40·04	2·95	–0·20, 6·11	0·06

* Single serve ≤ 500 ml; multi-serve > 500 ml.

On average, product prices in small shops were cheaper than they were in supermarkets in both periods. The relative price increase for taxed beverages was larger in small shops than in supermarkets (Figs 2 and 3) because baseline prices in small shops were lower.

**Fig. 1:**
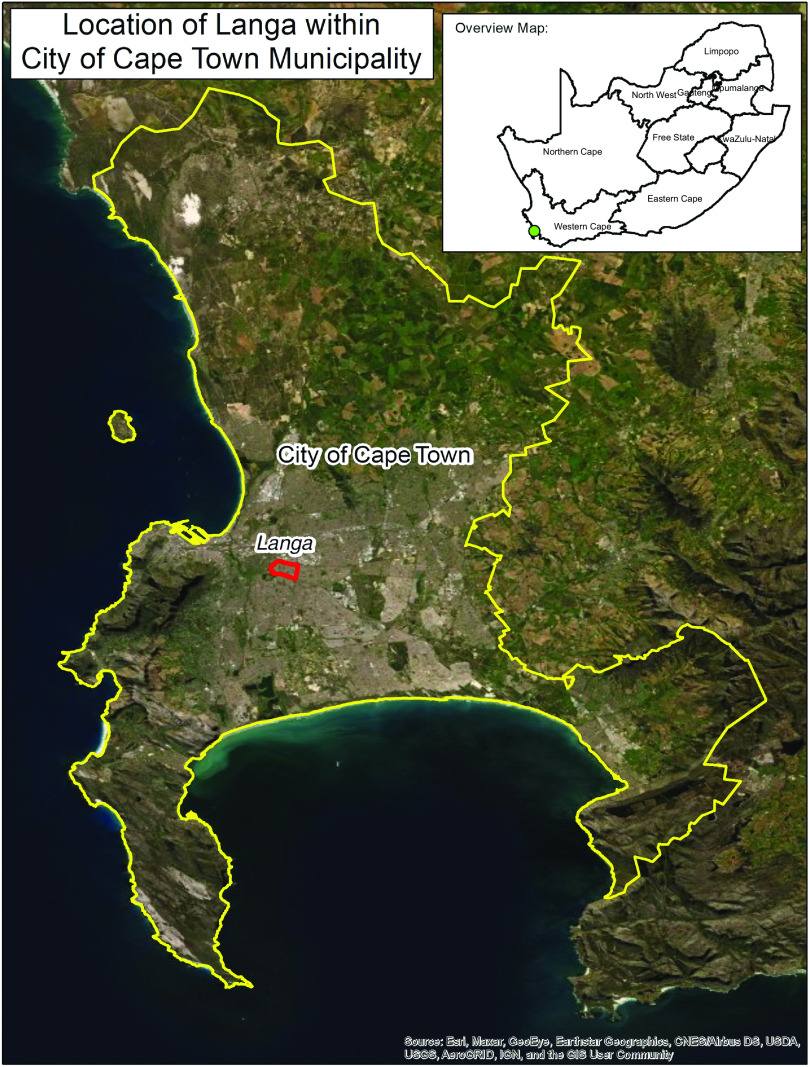
Map of the Western Cape. Supermarkets were located throughout the Western Cape, including one in Langa

**Fig. 2:**
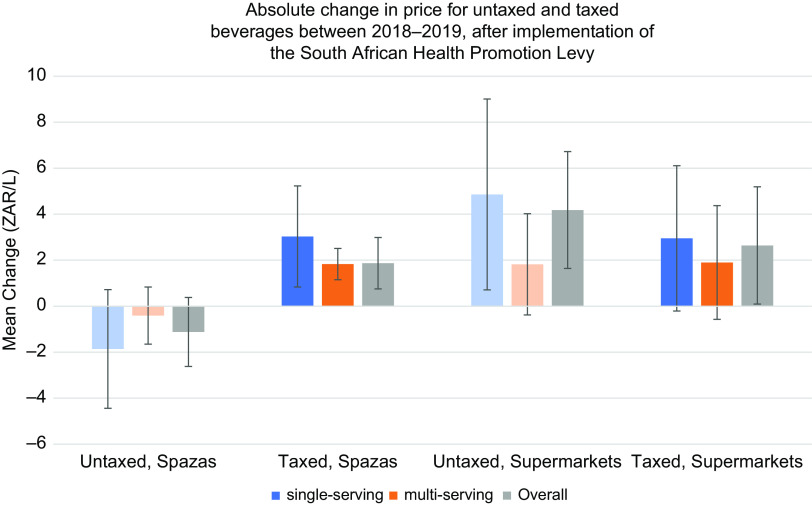
Absolute change in price for untaxed and taxed beverages in Supermarkets and Spazas between 2018 and 2019, pre/post-implementation of the South African Health Promotion Levy

**Fig. 3:**
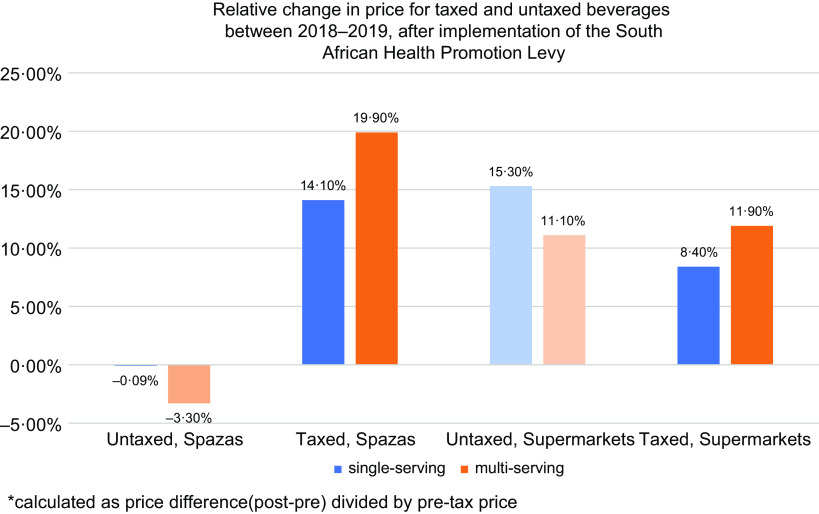
Relative change in price by tax status and store type between 2018 and 2019, pre/post-implementation of the South African Health Promotion Levy

### Acquisition

At baseline, 76 % of 18–39-year-old participants in Langa reported shopping at supermarkets, 62 % reported shopping at small shops and 5·5 % reported shopping at other independent retailers for beverages. Across all stores, there was a 9·1 percentage point decrease (CI: –12·5, –5·7) in the predicted probability of purchasing regular soda at least weekly between 2018 and 2019, after implementation of the tax (Table 3).

**Table 3:** Marginal effect of the HPL on post-period acquisition of select beverages at least once a week by store type among residents (age 18–39 years) living in Langa, Western Cape, South Africa, 2018–2019***

Beverage type	All		Supermarkets		Spazas		All other independent stores	
	Margins	95 % CI	Margins	95 % CI	Margins	95 % CI	Margins	95 % CI
Regular soft drinks	−0·091	–0·125, –0·057**	−0·103	–0·138, –0·067**	−0·121	–0·157, –0·085**	−0·039	–0·189, 0·111
Diet soft drinks	−0·065	–0·199, 0·070	−0·042	–0·184, 0·101	−0·055	–0·206, 0·096	†	
Milk and other dairy products	−0·106	–0·137, –0·076*	−0·107	–0·138, –0·077*	−0·118	–0·156, –0·081*	−0·184	–0·302, –0·067*
Coffee and tea	−0·017	–0·032, –0·002*	−0·019	–0·034, –0·005*	−0·015	–0·34, 0·004	†	

* *P*-value less than 0·05.

** *P*-value less than 0·001.

*** Models were adjusted for age, gender, BMI, number of people in the household and socio-economic status ascertained using the South African Audience Research Foundation’s Living Standards Measure (LSM). It is based on 29 items, including household income and education, and household assets, which were also added as covariates in the model. Other covariates included lived poverty index measures relating to availability of food and water, beverage consumption due to the drought and psychosocial measures relating to awareness of the HPL.

† *n* too small to run the model.

The marginal effect of the HPL from our adjusted model was a 10 percentage point decrease (CI: –13·8, –6·7) in probability of purchasing regular soda at least weekly in supermarkets, a 12 percentage point decrease (CI: –15·7, –8·5) in spazas and no statistically significant change in other independent stores in the post-implementation period. There was a decrease in the purchasing frequency for all beverages, although the decrease in weekly purchases of diet soda was not significant. Reductions in purchasing seem higher in small shops than supermarkets. However, standard errors for supermarkets and small shops overlap so they are not statistically different from each other.

## Discussion

This is the first study to evaluate the HPL in South Africa by looking at pricing and acquisition by store type. This is also the first study to evaluate the HPL in South Africa by looking at acquisition of beverages by a younger population living in a lower income area. These findings are not generalisable to the entire South African population, but are reflective of a lower income, younger adult population.

We found that the probability of purchasing regular soda weekly fell in both supermarkets and small shops, although no significant difference was observed in other independent stores. Purchasing also fell for untaxed diet sodas, although the differences between the pre- and post-tax implementation periods were not statistically significant. This may be because diet sodas were not a popular beverage at baseline and there were a low number of observations of purchasing of diet sodas in this context. Consistent with previous studies evaluating changes in purchasing in South Africa following implementation of the HPL using Kantar World Panel data^(30)^, we found that purchasing fell in every beverage category. Acquisition of untaxed dairy as well as coffee and tea also fell in this study, but not significantly. Our study found that in small shops, there was a non-significant change in price of untaxed beverages but a significant increase in prices of taxed beverages. These results are similar to a separate study by Stacey et al. which found null increases in price among tax-exempt products, and statistically significant increases in the prices of carbonates which are subject to taxation^(42)^. Conversely, there was an observed 4·86 ZAR/L increase in prices of untaxed single-serving beverages in supermarkets but not a significant increase in prices of taxed beverages. This observation could be a reflection of supermarkets or manufacturers strategically cost-shifting their increased costs across taxed and untaxed beverages and who they believe are more likely to purchase untaxed beverages.

From a public health viewpoint, the findings are indicative of an increase in the cost of consumption of taxed beverages relative to untaxed beverages in small shops. At the same time as the introduction of the HPL in April 2018, there was a 1 percentage point increase in the Value Added Tax for most food products from 14 % to 15 %. Although the increase is relatively small, it may matter for this population and may explain decreases in purchases of all beverages. Decreases in overall purchasing frequency could also be characterised independent of price, as it could be the result of increased media attention and conversation surrounding why the levy is being implemented that caused a decrease in purchasing^(43,44)^. Awareness of the tax might cause respondents to feel pressure to report lower beverage consumption (social desirability bias). However, HPL awareness was reported in the questionnaire and controlled for in the analysis.

Reductions in purchasing were greater in small shops than supermarkets. On average, prices of products (ZAR/L) in small shops are cheaper than they are in supermarkets in both periods, but the price increase on taxed products was comparable in both supermarkets and small shops. Given what we observed, the relative price increase was larger in small shops than in supermarkets because baseline prices in small shops were lower. This is in contrast to other studies in the United States that evaluated the tax by store type, where prices of taxed beverages in Berkley, Oakland, San Francisco and Seattle increased more in supermarkets than in convenience stores after implementation of their respective city’s SSB taxes^(33,45–47)^. In Philadelphia, larger tax increases were observed in pharmacies and small, independent stores than in supermarkets, although it is difficult to make direct comparisons to this South African context because prices in the pharmacies and small stores are higher than in supermarkets in Philadelphia or Baltimore^(48)^. In general, the finding that prices were lower in small shops than in supermarkets should be explored further. Small shop owners often purchase products from wholesalers and/or larger supermarkets, so it would be expected that they would be higher. However, it could be that special offers were given to small shops pre/post-HPL as a strategy of producers who changed the size of their containers in supermarkets to get rid of stock by selling cheaper to small shop cooperatives. Additionally, small shops only had a very small offering of products (a few of the most well-known brands and a greater selection of cheaper, local brands); whilst supermarkets had a vast range of higher end multi-national products, and only a few cheaper options.

We observed increases in the absolute and relative prices differences of untaxed beverages in supermarkets between 2018 and 2019. This is similar to findings in Berkeley where untaxed beverage prices increased in large supermarkets but lowered in independent corner stores^(49)^. This could suggest that in both locations, supermarkets are employing similar cost-shifting strategies to offset the increase in price of taxed beverages, which are not being utilised in small stores. Whether these are behaviors that are influenced by the manufacturers or retailers is unknown in the South African context because stocking in supermarkets and small shops was not captured, which makes it difficult to understand the scope of cost shifting in the stores that were evaluated. The HPL was levied on and collected from manufacturers/distributors and we do not know how much wholesalers charge so we did not have an opportunity to analyse cost shifting with this data. The scope of this study was to assess price change and beverage acquisition in one population, but future research should assess cost shifting, as it is important to see how retailers also respond to these taxes. Retailers’ responses have implications for the effectiveness of the tax for different populations and contexts.

This study had some additional limitations. Bottled water was not captured in the beverage acquisition instrument, so changes in its acquisition pre/post-implementation of the HPL were not assessed. However, the drought in Langa that persisted throughout the data collection period would have made these observations unreliable. Additionally, the acquisition instrument only captured 4 broad beverage categories (diet soda, regular soda, dairy, coffee and tea) and did not specify the brand or specific beverage purchased. This instrument was part of a larger questionnaire, so this was done for feasibility. This analysis relied on frequency of purchasing and would have benefitted from knowing the quantity participants purchased at each shopping trip, as this would affect the frequency of purchasing needed to stock the home. Another limitation is the lack of a geographic control or comparison city which would allow us to determine the increase in beverage price exogenous to factors unrelated to the tax. However, the HPL was a country-wide tax and evaluating pricing and acquisition of beverages in a different country with comparable characteristics would have been logistically challenging. Because of the sugar-density design of the tax, it is difficult to properly assess percent pass through, particularly since sugar reductions (due to manufacturer reformulation) occurred to some degree. However, work from other scholars that have evaluated the HPL in South Africa has shown that the overall pass through among carbonates (the largest contributor to SSB in South Africa) was approximately 68 %, with 100 % pass through on small containers and 50 % pass through on larger containers^(30,42)^. Lastly, pricing and acquisition data were not directly linkable because we did not know where participants specifically purchased their beverages and if they purchased them from one of the stores included in our dataset. However, this data were largely collected in the same community in the same time period, so many of the conclusions can still be made because it is likely that participants shopped in these and other similar stores. Additional strengths of this study include its large sample size which is comparable between time points, and use of the same months in comparisons to account for seasonality.

## Conclusion

After South Africa became the first SSA country to enact a sugary beverage tax, other countries on the continent are exploring similar fiscal policies, along with front-of-package labeling, to reduce the growing rates of SSB consumption and obesity^(50)^. Other SSA and low to middle-income countries are also battling the double burden of malnutrition, so it is important that interventions are implemented to increase food access while keeping high energy, low nutrient-dense foods at bay in this context. This work can add to that body of evidence to inform policies to improve this balance in the retail food environment in similar contexts and understand the implications of these policies in the retail setting in SSA and other low and middle-income countries.

## Supplementary material

For supplementary material accompanying this paper visit https://doi.org/10.1017/S1368980022000507. click here to view supplementary material
