# Supplementary material for: South Africa’s Health Promotion Levy on pricing and acquisition of beverages in small stores and supermarkets
Source: Public Health Nutr. 2022 Mar 7;25(5):1300–9. doi: 10.1017/S1368980022000507 (PMC9991735; doi:10.1017/S1368980022000507)
Supplement: Supplementary file 1 [file S1368980022000507sup001.docx]

**Appendix**

**Appendix 1: Example logit output for model describing purchasing of regular soft drinks at least weekly among people that shop at supermarkets and spazas**

|  | **Supermarkets** | | | | | **Spazas** | | | | |
| --- | --- | --- | --- | --- | --- | --- | --- | --- | --- | --- |
|  | Coef. | Std. Err. | z | P>\|z\| | [95% Conf. Interval] | Coef. | Std. Err. | Z | P>\|z\| | [95% Conf. Interval] |
| **age** | -0.0050 | 0.0077 | -0.65 | 0.515 | -.020107, .0100757 | -0.0118 | 0.0091 | -1.29 | 0.199 | -.0296653, .0061654 |
| **sex (female)** | -0.1485 | -0.1485 | -1.46 | 0.143 | -.3473089, .0502792 | -0.2383 | 0.1184 | -2.01 | 0.044 | -.470322, -.0061814 |
| **lsm_category** |  |  |  |  |  |  |  |  |  |  |
| **4** | -0.4744 | 0.3652 | -1.30 | 0.194 | -1.190086, .2413392 | -0.2984 | 0.4178 | -0.71 | 0.475 | -1.117316, .520565 |
| **5** | -0.1851 | 0.4188 | -0.44 | 0.658 | -1.005883, .6356939 | -0.1838 | 0.4847 | -0.38 | 0.705 | -1.133816, .7662052 |
| **6** | -0.3879 | 0.4970 | -0.78 | 0.435 | -1.362123, .5862293 | -0.5023 | 0.5777 | -0.87 | 0.385 | -1.634527, .6298388 |
| **year** | -0.4848 | 0.0882 | -5.50 | 0.000 | -.657638, -.3119034 | -0.6722 | 0.1044 | -6.44 | 0.000 | -.8768981, -.467526 |
| **bmi** |  |  |  |  |  |  |  |  |  |  |
| **18.5 – 24.9** | 0.1852 | 0.1889 | 0.98 | 0.327 | -.1850705, .5554134 | 0.1810 | 0.2158 | 0.84 | 0.402 | -.2419447, .6038922 |
| **25.0 – 29.9** | 0.1430 | 0.2022 | 0.71 | 0.479 | -.2532178, .5393006 | 0.1422 | 0.2316 | 0.61 | 0.539 | -.3117526, .5961069 |
| **> 30.0** | 0.2324 | 0.2058 | 1.13 | 0.259 | -.1709688, .6357982 | 0.3648 | 0.2370 | 1.54 | 0.124 | -.0997238, .8293765 |
| **drought** | 0.7639 | 0.1128 | 6.77 | 0.000 | .5427925, .9849732 | 0.8951 | 0.1395 | 6.42 | 0.000 | .6216404, 1.168484 |
| **washlocation** | -1.0703 | 0.1182 | -9.06 | 0.000 | -1.301935, -.8387483 | -0.9131 | 0.1377 | -6.63 | 0.000 | -1.182937, -.6432393 |
| **washtapwater** | 0.5430 | 0.1129 | 4.81 | 0.000 | .321768, .7641411 | 0.6099 | 0.1321 | 4.62 | 0.000 | .3509998, .8687277 |
| **childreninhousehold** | 0.0220 | 0.0323 | 0.68 | 0.496 | -.0413258, .0853853 | 0.0252 | 0.0441 | 0.57 | 0.568 | -.0612923, .1116751 |
| **employmentstatus** | 0.0016 | 0.0014 | 1.08 | 0.280 | -.0012736, .0044068 | -0.0005 | 0.0017 | -0.33 | 0.745 | -.003843, .0027482 |
| **hplawareness** | 0.3385 | 0.1180 | 2.87 | 0.004 | .1072494, .5696978 | 0.2378 | 0.1362 | 1.75 | 0.081 | -.029105, .5046918 |
| **numberinhousehold** | -0.0515 | 0.0269 | -1.92 | 0.055 | -.1041723, .0010967 | -0.0513 | 0.0311 | -1.65 | 0.099 | -.1122332, .0095745 |
| **foodsecurity** | 0.0161 | 0.0112 | 1.44 | 0.150 | -.0058292, .0380581 | 0.0073 | 0.0110 | 0.66 | 0.506 | -.0142619, .0288977 |
| **watersecurity** | -0.0033 | 0.0094 | -0.35 | 0.729 | -.0217334, .0151944 | 0.0013 | 0.0093 | 0.13 | 0.893 | -.0170712, .0195759 |
| **regular soft drink consumption** | 0.2973 | 0.0835 | 3.56 | 0.000 | .1335634, .4610263 | 0.4266 | 0.0982 | 4.35 | 0.000 | .2342292, .6190309 |
| **Household Assets:** |  |  |  |  |  |  |  |  |  |  |
| **hotwater** | 0.2967 | 0.9087 | 0.33 | 0.744 | -1.484277, 2.077592 | 0.2008 | 1.0331 | 0.19 | 0.846 | -1.824012, 2.225661 |
| **computer** | 0.7134 | 0.6713 | 1.06 | 0.288 | -.6023143, 2.029094 | 1.3090 | 0.7975 | 1.64 | 0.101 | -.2540754, 2.872124 |
| **electstove** | 0.0114 | 0.9839 | 0.01 | 0.991 | -1.917091, 1.939804 | 1.2816 | 1.0948 | 1.17 | 0.242 | -.8640821, 3.427349 |
| **domhelp** | 1.3058 | 2.9556 | 0.44 | 0.659 | -4.487025, 7.098533 | 2.6604 | 3.6780 | 0.72 | 0.469 | -4.548398, 9.869247 |
| **radio** | -1.1742 | 0.4809 | -2.44 | 0.015 | -2.116615, -.2317139 | -1.4663 | 0.5678 | -2.58 | 0.010 | -2.579106, -.3534228 |
| **toilet** | 0.1483 | 1.1286 | 0.13 | 0.895 | -2.063785, 2.360321 | -2.0184 | 1.3322 | -1.52 | 0.130 | -4.629514, .5927604 |
| **vehicle** | -3.3142 | 1.4608 | -2.27 | 0.023 | -6.177344, -.4510392 | -1.3208 | 1.8242 | -0.72 | 0.469 | -4.896263, 2.254625 |
| **frig** | -0.2978 | 0.9041 | -0.33 | 0.742 | -2.069782, 1.474269 | 0.6862 | 1.0216 | 0.67 | 0.502 | -1.31616, 2.688498 |
| **vacuum** | 2.6787 | 2.9617 | 0.90 | 0.366 | -3.126213, 8.483567 | 0.7177 | 3.2077 | 0.22 | 0.823 | -5.56923, 7.004688 |
| **paytv** | 1.1873 | 0.8609 | 1.38 | 0.168 | -.5000415, 2.874626 | 1.0333 | 1.0284 | 1.00 | 0.315 | -.9823933, 3.048994 |
| **dishwasher** | 1.6916 | 1.5866 | 1.07 | 0.286 | -1.418004, 4.801185 | 3.2937 | 2.2422 | 1.47 | 0.142 | -1.100985, 7.688452 |
| **cellphone3** | 0 (omitted) |  |  |  |  | 0 (omitted) |  |  |  |  |
| **cellphone2** | 0 (omitted) |  |  |  |  | 0 (omitted) |  |  |  |  |
| **homesecurity** | -5.4929 | 5.5323 | -0.99 | 0.321 | -16.33602, 5.350205 | -0.2403 | 5.7279 | -0.04 | 0.967 | -11.46684, 10.9863 |
| **freezer** | 1.9530 | 1.9538 | 1.00 | 0.317 | -1.87629, 5.782309 | 0.0221 | 2.1950 | 0.01 | 0.992 | -4.280017, 4.324247 |
| **microwave** | -0.8873 | 0.8373 | -1.06 | 0.289 | -2.528393, .7538239 | -0.1868 | 0.9784 | -0.19 | 0.849 | -2.104386, 1.73082 |
| **dvd** | -0.9774 | 1.0504 | -0.93 | 0.352 | -3.03613, 1.081364 | -0.7084 | 1.2366 | -0.57 | 0.567 | -3.132044, 1.715252 |
| **dryer** | 3.8587 | 2.2643 | 1.70 | 0.088 | -.5792851, 8.296708 | 5.8768 | 2.9755 | 1.98 | 0.048 | .0448471, 11.70867 |
| **hometheatre** | 0.0011 | 2.6829 | 0.00 | 1.000 | -5.257245, 5.259487 | 1.0870 | 3.2111 | 0.34 | 0.735 | -5.20668, 7.380655 |
| **homephone** | -6.2704 | 3.7216 | -1.68 | 0.092 | -13.56461, 1.023906 | -4.8426 | 4.2626 | -1.14 | 0.256 | -13.19725, 3.511993 |
| **pool** | 0 (omitted) |  |  |  |  | 0 (omitted) |  |  |  |  |
| **tapwater** | -0.8831 | 11.0570 | -0.08 | 0.936 | -22.55437, 20.78825 | -3.6916 | 10.3226 | -0.36 | 0.721 | -23.9235, 16.54025 |
| **sink** | 3.9204 | 0.9336 | 4.20 | 0.000 | 2.090543, 5.750207 | 2.1755 | 1.0795 | 2.02 | 0.044 | .0598207, 4.29121 |
| **tvset** | -1.6982 | 1.1885 | -1.43 | 0.153 | -4.027577, .6311431 | -0.5370 | 1.3615 | -0.39 | 0.693 | -3.205473, 2.131373 |
| **ac** | -2.1085 | 2.0221 | -1.04 | 0.297 | -6.071596, 1.854696 | -1.5408 | 2.3722 | -0.65 | 0.516 | -6.190128, 3.10857 |
| **ses_wt_total** | -0.8544 | 0.4503 | -1.90 | 0.058 | -1.736869, .0281623 | -0.2471 | 0.5089 | -0.49 | 0.627 | -1.244582, .7503254 |
| **constant** | 980.3656 | 178.1040 | 5.50 | 0.000 | 631.2882, 1329.443 | 1359.7980 | 210.8669 | 6.45 | 0.000 | 946.5063, 1773.089 |
